# Review

**Published:** 1864-07

**Authors:** 


### REVIEW.

*Bumstead on Venereal Disease.*

We have received the second edition of this valuable work, we should rather say invaluable work, for it is the only systematic treatise so far as we know which ought to have the authority of a guide in the study and treatment of syphilis. It is one of the most important, if not the most important work which has been offered to the profession. When we consider the erroneous notions which were held before its appearance by the majority of the profession; when we take into account the consequences involved in correct knowledge, or we had better say want of correct knowledge of the subject of which it treats, the above commendation does not seem at all extravagant. Besides that it clears up thoroughly the experience of good and careful observers, it enables them to lay aside many doubts as to methods of treatment, and to give more distinct and definite assurances to patients. The clear and undoubted distinction which is drawn, between the chancre and the chancroid, i. e. between the infecting and the non-infecting chancre, will not only, in most cases, enable the surgeon to make a certain diagnosis, and hence proceed understandingly in the treatment of them, but will afford him the means of relieving patients from that distressing doubt and apprehension which in many cases weigh so heavily.

This edition shows some changes, consisting of additions from the most recent investigations; of taking away, as in the chapter on syphilization, which is considerably lessened both in matter and importance; and of arrangement. Syphilization which seemed always so repugnant, now appears in its true light, simply as a measure which by its depurative action upon the system, leads to apparent benefit in the treatment of syphilis. As first proposed, as a prophylactic against syphilis, it certainly seemed rather shocking, though, of course, had there been any real truth at the bottom of it, scientific men would have been compelled to follow whithersoever it might lead. When we consider that the virus used was always that of the chancroid, which is followed by no constitutional infection, we can see how absurd was the idea, and also that whatever benefit follows the repeated inoculation of the same virus in cases of true syphilis, can only be purely and simply depurative.

The arrangement in this edition is much more satisfactory than in the first, viz: an entirely separate consideration of the chancre and the chancroid. Their consideration together was perhaps a necessary concession to the doubts of the profession, at the time the first edition was issued. No such necessity now exists, since the new doctrines put forth concerning them are very generally accepted.

The work is divided into three parts. In the first the author treats ably and fully of gonorrhœa and its complications. We do not propose to say more of this than that the subject is as thoroughly and clearly treated as in any existing work, and we may say more fully up to the times. The subject of stricture of the urethra, is treated in a full and satisfactory manner. All methods of dealing with this often perplexing disease, which have been found to have any value, receive due attention, among them the "immediate plan" of Mr. Holt, which commends itself to our judgment as one likely to be of more service than any yet proposed. It secures the best results of dilatation at less expense of time. The second part discusses the chancroid and its complications. The third part treats of syphilis. Nothing essentially new, we believe, has been added in regard to the difference between chancre and chancroid, though their discussion in separate chapters is not only proper, but aids a thorough recognition of the broad distinction between them.

We have then, in this important treatise, what we have nowhere else, in any work with which we are acquainted, the two kinds of sores on the genitals classified, discussed, and in topical arrangement, placed separately, as being distinct—wholly different in their nature, in their consequences, and in the measures of treatment to which they should be subjected. We wish to call attention to the prominence given to the suppurating bubo, as a means of diagnosis. It may be taken for granted, in almost all instances, that when there has been or is, a suppurating bubo in the groin in connection with a sore on the genitals, there neither has been, nor will be constitutional infection, and therefore no need of specific treatment, or any other than local. We must admit that all cases are not capable of an exact diagnosis. There are certain mixed cases, in which symptoms of both chancre and chancroid are present. These cases are however few. For the formation and giving of a correct opinion exactness is desirable, but as far as treatment is concerned, it matters little, if the correct practice is only to treat syphilitic manifestations when they appear.

In regard to treatment, it will be observed that the author gives in this edition, his preference to the external use of mercury, both in the form of bath and inunction, the latter being an excellent method in all respects, except its want of cleanliness. Used in these ways, the effect of mercury is more rapid, and less irritating to the digestive organs, and less likely to cause troublesome salivation. We think the discussion of the action and benefit of mercury, and the reasons for choosing one form rather than another, in fact the directions given for the use of the drug, is particularly full, definite and satisfactory. We notice that the author differs from Dr. Dixon, whose recommendations are generally sound and practical, in regard to the importance of dilating the pupil by mydriatics in the treatment of iritis. He insists that it is an essential measure, in which we fully concur with him. It should be added that the author favors the withholding of mercury till some manifestations of general syphilis other than the chancre appear. The belief that mercury will prevent the secondary manifestations of syphilis, has been so general, that such a recommendation will, we think, be adopted with hesitation. When the belief prevails, as it should, that these manifestations are governed by a law, and not controlled by medicine, the plan of preventing secondary symptoms by mercury will fall into disuse. One thing, which we have not heretofore admitted, we may learn, viz: that nature has some power to eliminate the poison of syphilis. Therefore we must trust to hygienic influences as well as medicinal.

It is difficult to overstate the value of this work to the general practitioner. A careful study of it will save him, in numberless instances, from that needless use of mercury in many obscure cases, upon a vague suspicion that there may be something "specific" about them. When he has once settled in his mind, the general truth, which the book under notice so convincingly presents, that syphilis has pretty definite laws of manifestation, unmistakable, and for the most part constant, he will not grope in the dark, to feel out, as it were, by the use of mercury a "specific" taint. Let him consider this, and the fact, that of ten sores on the genitals, eight are generally chancroids, needing no specific treatment; and he will often have occasion to feel thankful for so valuable a guide as Dr. Bumstead has furnished him. But though cases of true syphilis are really more rare than was formerly supposed, let no one think lightly of it. This limitation only serves to show what a terrible disease syphilis is. It thoroughly infects the blood, there being no escape, and often runs a most destructive course in spite of our measures.

In conclusion, we may say, that no man ignorant of the facts and doctrines of the book before us, has any right to undertake the treatment of venereal sores. The book is one which every practitioner should have in his possession, and we may further say, the *only* book upon the subject which he should acknowledge as competent authority.

---

*The Principles and Practice of Obstetrics: Illustrated with One Hundred and Fifty-Nine Lithographic Figures, from Original Photographs, and with numerous Wood-Cuts.* By Hugh L. Hodge, M. D., *Emeritus Professor of Obstetrics and Diseases of Women and Children, in the University of Pennsylvania; lately one of the Physicians to the Lying-in Department of the Philadelphia Hospital; lately one of the Physicians to the Philadelphia Almshouse Hospital; Consulting Physician to the Philadelphia Dispensary; Fellow of the College of Physicians of Philadelphia; Member of the American Philosophical Society, etc.; Author of a treatise on "The Peculiar Diseases of Women."* Philadelphia: Blanchard & Lea. 1864.

It would at first seem that there was really nothing to be added to our numerous and valuable works on obstetrics—that everything known of the art had been embodied in several of the recently published works upon this subject. An examination of this book will, however, convince us of this error, and show how wide is this field, how actively it is being cultivated, and how much yet remains to be discovered. In this volume, the author has presented not simply his own opinions, but also those of the most distinguished in the profession, so that it is a digest of the theory and practice of obstetrics.

This branch of medical practice had received but little attention, or had made but little progress, until within the last fifty years, and the appearance of such a volume shows how much has been accomplished during that short period. The preface contains, with a great many other things, a history of obstetrics in the United States, in which it is stated that at the commencement of the last century obstetrics in the United States was regarded altogether as a subordinate branch of medicine; its practice was entrusted to women, and it was only in cases of tedious and dangerous labors, that the assistance of the surgeon was required. Obstetrics has not been taught in our schools as a distinct branch of medical practice until within a comparatively short period. Harvard University, in Cambridge, Massachusetts, in its arrangements of professors, did not bestow any special attention upon obstetrics until as late as 1815, when Dr. Walter Channing was appointed Lecturer upon Obstetrics and Medical Jurisprudence. We must not follow this history, though it is exceedingly interesting and shows how great improvements in this department have been made within the last half century.

Great labor has been bestowed upon the preparation, and great expense incurred, in the publication of this work. It is astonishing that such an extensive and complete treatise upon any branch of medical practice should appear at such a time, and shows that the medical profession of America are anxious to be in possession of all that is known upon the subject. We have no work in our language so extensive and complete upon the art and practice of obstetrics; and though several works of great merit have appeared within the last few years, still, no medical library can be considered complete without this volume,

The illustrations are numerous and complete; as an illustrated work, it is superior to any which has appeared. We should be glad to give a list of the subjects represented, but suffice it to say that everything desirable has been faithfully and naturally represented by lithographic plates, prepared with great accuracy and care. On this account, as a textbook for the student and young practitioner, it is of incomparable value.

For sale in Buffalo by THEODORE BUTLER.

